# Economic Impact of Maternal Death on Households in Rural China: A Prospective Cohort Study

**DOI:** 10.1371/journal.pone.0076624

**Published:** 2013-10-24

**Authors:** Haijun Wang, Fang Ye, Yan Wang, Dale Huntington, the study group for Economic Impact of Maternal Deaths in China

**Affiliations:** 1 Division of Maternal and Child Health, School of Public Health, Peking University, Beijing, China; 2 Asia Pacific Observatory on Health Systems and Policies, World Health Organization Western Pacific Regional Office, Manila, Philippines; University of Alabama at Birmingham, United States of America

## Abstract

**Objective:**

To assess the economic impact of maternal death on rural Chinese households during the year after maternal death.

**Methods:**

A prospective cohort study matched 183 households who had suffered a maternal death to 346 households that experienced childbirth without maternal death in rural areas of three provinces in China. Surveys were conducted at baseline (1–3 months after maternal death or childbirth) and one year after baseline using the quantitative questionnaire. We investigated household income, expenditure, accumulated debts, and self-reported household economic status. Difference-in-Difference (DID), linear regression, and logistic regression analyses were used to compare the economic status between households with and without maternal death.

**Findings:**

The households with maternal death had a higher risk of self-reported “household economy became worse” during the follow-up period (adjusted OR = 6.04, *p<*0.001). During the follow-up period, at the household level, DID estimator of income and expenditure showed that households with maternal death had a significant relative reduction of US$ 869 and US$ 650, compared to those households that experienced childbirth with no adverse event (*p<*0.001). Converted to proportions of change, an average of 32.0% reduction of annual income and 24.9% reduction of annual expenditure were observed in households with a maternal death. The mean increase of accumulated debts in households with a maternal death was 3.2 times as high as that in households without maternal death (*p = *0.024). Expenditure pattern of households with maternal death changed, with lower consumption on food (*p = *0.037), clothes and commodity (*p = *0.003), traffic and communication (*p = *0.022) and higher consumption on cigarette or alcohol (*p = *0.014).

**Conclusion:**

Compared with childbirth, maternal death had adverse impact on household economy, including higher risk of self-reported “household economy became worse”, decreased income and expenditure, increased debts and changed expenditure pattern.

## Introduction

Maternal health has understandably become a focus of world attention as we approach2015, the target date for the Millennium Development Goals (MDGs). As the biggest developing country in the world, China bears a heavy burden of maternal mortality as one of 74 countdown countries to achieve MDG5 [1]. Increasingly studies conducted in different countries and regions worldwide show that the poor are more vulnerable to maternal death than the richer segment of the population [2], [3]. Additionally, poorer households are challenged by maternal death with its double jeopardy of catastrophic costs and diminished productivity, for maternal deaths usually occur in women's prime productive years [4], [5]. However, this topic has not been widely explored in the literature on maternal health. We did a systematic search on electronic databases of Pubmed, Embase, PAHO and Popline, and on home pages of major international organizations for published literature. We identified only one publication, which was based on the baseline results of our study and described the immediate but not lasting economic effects of maternal deaths on rural Chinese households [6]. The average direct costs of maternal death was up to US$ 4,119, and the economic burden, which referred to the ratio of out-of-pocket costs to total household annual income, was 37.0%, being approximately four times as high as the threshold for being considered catastrophic [6]. In recent years, emergency obstetric care have been found to be a useful complement to investigation of maternal mortality, and a few studies have estimated the impact of these cases on household economy [7]–[9]. However, knowledge is still lacking about the precise long-term impact of maternal death on household economy.

Until now in current health insurance system in China, there are three major ways to protect rural pregnant women from economic burden of maternal care utilization. In 2000, Chinese government launched the National Program to Reduce Maternal Mortality and Eliminate Neonatal Tetanus in rural areas, one task of which is to offer subsidies to women for paying institutional delivery [10], [11]. Three years later, the government carried out the New Cooperative Medical Scheme (NCMS), which provide reimbursement to women that delivered in hospital [12]. The reimbursement policy is decentralized and varies by county: in general vaginal deliveries are reimbursed for a flat fee and caesarean sections are reimbursed as a proportion of total expenditure within a set ceiling [13]. In 2009, the government launched the Institutional Delivery Subsidy Program to the whole rural areas of China, which provide financial assistance for each case of institutional delivery. These existing programs aimed to increase institutional delivery. However, until now, there is no policy or program to support poor rural families who have suffered a maternal death, even though this has been demonstrated as having immediate catastrophic impact on household economy with very little financial assistance from government or society [6]. Lack of financial assistance is in part due to gaps in the evidence base on the sustained effects on the household economy.

It is well accepted that longitudinal study design is the better way to establish causal relationship rather than the cross-sectional survey at a single point of time. Despite great difficulties and high costs for obtaining follow-up information of those households suffering from maternal death, prospective surveys in the same cohort are needed to track changes over time for clarifying the relationship between maternal death and household economy [5]. Therefore, we performed this prospective cohort study among households with maternal death (the affected group) and households of childbirth without maternal death (the comparison group) in rural areas of China. We analyzed the data from the baseline survey (having maternal death or childbirth within 3 months) and follow-up surveys (one year after baseline survey) on the same cohort by comparing changes of household economic indicators between the affected and comparison groups, including income, expenditure, debts, and self-evaluation of the overall impact of maternal death. The study aimed to identify whether there was the economic impact of maternal death on households in rural China and how serious it was.

## Participants And Methods

This is a prospective cohort study, involving 183 households experiencing maternal death and 346 households of childbirth without maternal death. The households were located in rural areas of three provinces (Hebei, Henan, and Yunnan), which were purposively selected to represent settings with low, moderate, and high MMRs in rural China.

### Participants

According to the inclusion criteria listed below, potential eligible households were identified by the County Maternal and Child Health Office in the selected provinces, which routinely collects information of every case of maternal deaths in the county no matter whether it happens in health facilities or not. After eligibility was confirmed and informed consent was made, the person in charge of the household economy was invited to a face-to-face, standardized interview. The comparison households were identified according to the matching criteria.

Inclusion criteria for the households with maternal death:

- Having maternal death within 3 months of the baseline interview; Maternal death defined as the death of a woman while pregnant (≥28 weeks gestational age) or within 42 days of termination of pregnancy, not for accidental causes unrelated to the pregnancy.

Matching criteria for the households of childbirth without maternal death:

- Having childbirth within 3 months of the baseline interview, living in the same administrative village, with similar economic status evaluated by the administrative village cadres (i.e. rich, moderate, poor), and the household type is the same as that of the affected family before maternal death (i.e. nuclear or extended; with or without older children).

A total of 530 cases of maternal death were identified in Hebei, Henan, and Yunnan provinces between June 2009 and October 2010. Of these cases, 40.9% were determined to ineligible or did not meet the inclusion criteria (217 out of 530) and an additional 22.3% of the cases (118 out of 530) were eligible but refused to be interviewed. The principle reasons for refusing to be interviewed were unresolved medical malpractice legal suits, or extreme grief among the surviving family members. Overall, 36.8% of the total number of maternal deaths were eligible and accepted the baseline interview (195 out of 530). We compared the gestational week and age of maternal deaths between those accepted and refused to be interviewed. No significant difference was found (data not shown). We selected 384 matching households of a childbirth without maternal death according to selection criteria. Six of the affected households were matched with only one comparison due to difficulties in identifying a second eligible comparison family in the remote and under-populated areas. We compared the women's age and gestational week of maternal deaths between those accepted and refused to be interviewed. Results showed that no significant difference was observed (data not shown).

Among households that enrolled in baseline study, 183 affected households (93.8%) and 346 comparison households (90.1%) accepted the follow-up survey at one year after their intake interview. The attrition rate was 6.2% in affected group and 9.9% in the comparison group. The reasons for attrition in affected group included “unavailable for off-farm employment” (6 cases), “unavailable for traffic inconvenience” (4 cases), “refused follow–up survey” (1 case), “moved to another place” (1 case). The reasons in comparison group included “not visiting the comparison households matched for the above 12 affected households lost in follow-up survey” (24 cases), “unavailable for off-farm employment” (11 cases), “moved to another place” (3 cases). We compared the maternal and household characteristics (age, parity, ethnicity, occupation of woman, household size, whether the family was nuclear family, and dependency ratio) of the 183 affected households having both baseline and follow-up data and the 12 affected households only having baseline data. Results showed that no significant difference was observed (data not shown).

### Data collection

The baseline survey data were collected between June 2009 and October 2010 and the follow-up surveys were conducted at one year after the baseline interview for each respondent. During each interview, we collected data with quantitative questionnaires designed after a literature review of related studies, expert consultation, ethical review and pilot study.

The questionnaire included questions on the maternal and household demographic status, household economic status, and health status. Maternal and household demographic status included age, parity, ethnicity, and occupation of woman, household size, whether the family was nuclear family, etc. Household economic questions included both subjective and objective indicators. The subjective indicator was self-evaluation of the overall impact of maternal death, i.e. whether their household economy “became worse” in the following year after baseline survey, and the objective indicators were household and individual income, expenditure and debts. In the questionnaire, there were 31 items of income, including salary (1 items), family production income (27 items, produced gain, slaughtered pigs, output of milk, etc.), and income from transfer and estate (3 items, gifts, donations and other forms of transfer from community members). There were 47 items of expenditure, including expenditure of production (9 items, agricultural productive expenditure, livestock expenditure, etc.), living expenses (30 items, food, clothes and commodity, residence, etc.), and expenditure for transfer and estate or tax (8 items). In order to make the annual household income and expenditure comparable in the year before and after the event of maternal death or childbirth, we excluded enormous occasional income or expenditure in calculating annual household income or expenditure, which include cash as gift from relatives or friends for marriage or funeral, or high resettlement subsidies from government, expenditures for wedding or funeral ceremony, etc. In the follow-up survey, we collected data of expenditure for raising the survived newborn after maternal death or childbirth, which was also not included in total annual household expenditure, for this part of expenditure was US$ 0 in the year before maternal death or childbirth.

The baseline survey collected economic data during the year before maternal death or childbirth, while the follow-up survey collected data during the year since the baseline survey interview. Information collected in this study was based on recall of participants. For comparing annual income at individual level derived from our study with national public data, we collected data of rural net income per capita published by National Bureau Statistics [14], [15].

### Ethics Statement

The study protocol and data collection instruments were approved by the ethical reviews boards of Peking University, and the World Health Organization. Signed informed consent statements were obtained prior to any interview by the person in charge with household economy, and other family members who also participated in the interview. For one woman whose age was under 18, we obtained written informed consent from herself, her parents and husband. All the interviews were entirely voluntary (no incentives were provided).

### Data analyses

The database was developed using the Epidata 3.1 software. Statistical analyses were conducted with SPSS Version 15.0. The demographic characteristics (age, parity, ethnicity, and occupation of woman, household size, whether the family was nuclear family and dependency ratio) were compared between the affected and comparison group. For categorical variables, χ2 or Fisher's exact tests were used for comparison. For continuous variables, difference in means for normally distributed data was tested by t-test, and other continuous variables which had skewed distribution were tested by rank-sum test. *p<*0.05 was considered to be statistically significant. The average exchange rate in 2011 (US$1 = ¥6.5) was used to convert the money from Chinese RMB (¥) to US$. All income, expenditure, and debt data in the following year after baseline survey were adjusted by the average deflator of 5%.

We conducted logistic regression analysis with backward method to evaluate the subjective estimator of the overall impact of maternal death, analyzing self-reported “household economy became worse” in the following year after baseline survey. The model is *logit (P_i_) =  β_0_+β_1_X_1_+β_2_X_2_*, in which the dependent variable *P_i_* was a dichotomous variable indicating whether the household self-reported “household economy became worse”, while *β_1_* was the effect of maternal death, *β_2_* was the term of vector of covariates which included maternal and household demographic characteristics (age, parity, ethnicity, and occupation of woman, household size and whether the family was nuclear family, etc).

We assessed both “absolute value” and “relative value” of objective estimator of economic status. We used difference-in-difference (DID) approach to evaluate the “absolute value” [16], i.e. the average effects of maternal death on household and individual annual income, expenditure, or debt. Individual data was derived by dividing total household data by household size. We excluded the newborn from household size to eliminate the confounding effect caused by the survival status of newborn in two groups. Our cohort data were analyzed with one form of DID model, ΔY_it_ = A_i_ *a+Δe_it_, in which *Y_it_* refers to income, expenditure, or debt data of each household during the year before baseline or follow-up survey. *ΔY_it_* equals to the change of annual income, expenditure, or debt of each household between the two years. *A_i_* refers to whether there was maternal death. *a* is the effect of maternal death we were interested in, and *e_it_* is the residual error. All time-invariant household and individual characteristics which may correlate with the economic indicators were controlled during the process of “differencing” within each group. DID estimators for income, expenditure, and debt were shown in the results.

We then evaluated the “relative value” of the objective estimator of economic status by calculating changes of annual income and expenditure in terms of proportions. Linear regression models with covariance analysis was used, that is, with logarithmically transformed follow-up values as outcomes and respective logarithmically transformed baseline values as explanatory variables. All the income and expenditure data was transformed because of their positively skewed distributions. The antilog of regression coefficient represents the ratio between affected and comparison group [17]. The difference between the ratio and 1 equals the change of annual income and expenditure, and is converted into proportions by multiplying 100.

## Results

### Demographic characteristics of the affected and comparison groups

Table 1 shows the demographic characteristics of the affected and comparison group before maternal death or childbirth. The two groups did not differ in the proportions of primiparas (*p = *0.263), minority (*p = *0.255) and peasants or housewives (*p = *0.673). Women in the affected group tended to be older (29.5 vs. 26.3, *p<*0.001) than women in the comparison group. Before maternal death or childbirth, the characteristics of affected households were similar to comparison households, including household size (*p = *0.884), proportion of nuclear families (*p = *0.809), and dependency ratio (*p = *0.329).

**Table 1:** Demographic profile of the affected and comparison groups at baseline survey.

	Affected group	Comparison group	P
Sample size	183	346	-
Demographic characteristics of the women before maternal death or childbirth			
Median age (Range in Years)	29.5 (18.3–45.1)	26.3 (17.4–42.5)	<0.001
% of primiparas a	36.6	41.6	0.263
% of minority b	25.1	20.8	0.255
% of peasants or housewives	86.9	85.5	0.673
Demographic characteristics of the household before maternal death or childbirth			
Household size (Mean ± SD)	4.5±1.4	4.5±1.6	0.884
% of nuclear families	32.2	31.2	0.809
Dependency ratio c (Mean ± SD)	0.4±0.5	0.3±0.3	0.329

Affected group: households with a maternal death; Comparison group: matched households without maternal death; SD: Standard Deviation.

a Primiparas refers to a woman who has given birth once.

b In China, “minority” means non-Han ethnicity, including Hui, Yi, Hani, etc.

c Dependency ratio is the ratio of dependent part (number of those under the age of 15 and over the age of 64 in the household) to productive part (number of those ages between 15–64 in the household).

### Self-evaluation on the overall impact of maternal death

Sixty-three percent of the affected households and 29.5% of the comparisons self-reported “household economy became worse”, with statistically significant difference (*p<*0.001). Univariate logistic regression showed that the affected group carried significantly higher risk of worsening household economy (Odd ratio (OR)  = 4.07, 95% CI: 2.78–5.95, *p<*0.001). We conducted multiple logistic regression by controlling whether the newborn survived, whether the husband remarried, and maternal and household demographic characteristics which were listed in Table 1, including the women's age, whether the woman was primiparas or not, ethnicity (whether she was minority or not), occupation (whether she was peasants or housewives or not), household size, household structure (whether the household was nuclear family or not), and dependency ratio. The results indicated an increased OR (6.04, 95% CI: 3.61–10.08, *p<*0.001) of maternal death on worsening household economy, and none of the controlled independent variables were statistically significant except the survival of the newborn (OR = 2.26, 95% CI: 1.20–4.25, *p* = 0.012).

### Total annual income and expenditure

We excluded enormous occasional income or expenditure in the calculation of total income or expenditure (as previously described in methods of data collection). As shown in Table 2, the DID estimator of average annual income at household level showed a statistically significant difference between two groups (US$ –869, *p<*0.001). Average annual income per capita (at individual level) in both groups increased during the following year after baseline survey. Its DID estimator showed that the increase in the affected group was US$ 102 lower than that in the comparison group significantly (*p = *0.038). Converted to proportion of change, a 32.0% reduction of average income at household level and 19.9% at individual level was observed between the two groups. Excluding the expenditure for raising the newborn and enormous occasional expenditures, the average annual household expenditure was reduced significantly during the year after baseline survey in affected group (US$ –575, *p<*0.001), but almost was unchanged in the comparison group (US$ 74, *p = *0.429). Similar to annual income, the DID estimator of average annual expenditure at household level showed a statistically significant decrease in affected group (US$ –650, *p<*0.001). At individual level, average annual expenditure slightly decreased during follow-up period in the affected group (US$ –41, *p = *0.242), while it increased significantly in the comparison group (US$ 39, *p = *0.047). DID estimator showed that the mean relative difference between two groups was US$–80 (*p = *0.047). Converted to proportion of change, a 24.9% reduction of expenditure at household level and 12.4% at individual level was observed between the two groups.

**Table 2:** The results of difference-in-difference analyses (DID) for indicators of economic status between affected and comparison households (Mean, US $).

		Annual income		Annual expenditure		Cumulated debts	Household wealth indicators		
		household	per capita	household	per capita		Index score of family property d	Index score of live stocks e	Index score of basic living ability f
Affected group	Baseline	2862	657	2843	652	2421	2.68	2.51	5.60
	Follow-up	2807	761	2268	610	3762	2.68	1.86	5.55
	Δ_affected 1_ a (*p*-value)	−55 (0.715)^c^	104 (0.004)	−575 (<0.001)	−42 (0.268)	1341 (<0.001)	0.00 (0.879)	−0.65 (0.019)	−0.05 (0.463)
Comparison group	Baseline	3723	838	3211	738	1956	2.91	3.28	5.77
	Follow-up	4538	1044	3285	776	2375	2.98	2.82	5.82
	Δ_comparison_ b (*p*-value)	815 (<0.001)	206 (<0.001)	74 (0.429)	38 (0.047)	419 (0.002)	0.07 (0.382)	−0.46 (0.138)	0.05 (0.069)
DID estimator c	Δ_affected1_- Δ_comparison_ (*p*-value)	−870 (<0.001)	−102 (0.038)	−649 (<0.001)	−80 (0.053)	922 (0.024)	−0.07 (0.249)	−0.19 (0.697)	−0.10 (0.006)

Affected group: households with a maternal death; Comparison group: matched households without maternal death.

a Δ_affected_ refers to the mean value of affected group in follow-up minus that at baseline.

b Δ_comparison_ refers to the mean value of comparison group in follow-up minus that at baseline.

c Numbers in parentheses are *P*-value.

d 1 score for each item with answer “have”, 0 score for each item with answer “don't have”. All 5 items were summed up. The items included (1) TV, (2) Cable TV, (3) Telephone, (4) Motorcycle, electrobicycle, or elctrotricycle, (5) Truck, tricar, or tractor.

e All 3 items were summed up. The items included (1) How many pigs are there in your family? (2) How many sheep is there in your family? (3) How many other live stocks are there in your family, such as cattle, horse, donkey, mule, etc?

f 1 score for each item with answer “yes”, 0 score for each item with answer “no”. All 6 items were summed up. The items included (1) Can your family have enough food to be not hungry? (2) Can your family eat meat, egg, or fish at least once every week? (3) Can your family buy enough clothes to keep warm in winter? (4) Can your family buy a piece of new clothes? (5) Can your family members go to the hospital when having indisposition? (6) Can your family buy some daily necessaries (e.g. Toilet paper, shampoo, soap, washing powder, etc)?

Although annual income in both groups probably covered all expenditure in the year (as shown by the means of income and expenditure), the average accumulated debt increased much higher in affected group compared to that in comparison group (DID estimator =  US$ 922, *p = *0.024). The self-reported reasons were that households needed money to cover extra expenditure of raising the newborns or unexpected occasional event. In the affected group, the mean expenditure for raising the newborn (102 cases with survived newborns) was as high as US$ 1007 (inter-quartile range US$ 417 to US$ 1436), for 100.0% of them having bottle or formula feeding instead of breastfeeding. Besides, the mean expenditure for remarriage of the husbands in affected group (26 cases) was as high as US$ 4667(inter-quartile range US$ 934 to US$ 6081).

We also analyzed household wealth indices, including index score of family property, live stocks, and ability to maintain basic living including enough food, clothes, medical care, etc. The DID estimators of index score of family property and basic living ability showed significantly decrease in the affected group compared with that in the comparison group (−0.13, *p = *0.029; −0.10, *p = *0.006).

Since newborn survival associated with increased OR for self-reported “household economy became worse”, we did stratified analyses for quantitative indicators by whether the newborn survived or not. As shown in Table 3, affected households with or without newborn survival was associated with the same trend of reduced DID estimates of total income and expenditure in the year since the baseline, at both household and individual level. They also had increased DID estimates of cumulated debts, and reduced DID estimates of other household wealth indicators. Some estimates were not statistically significant, which may be related with insufficient power of 0.561 for subgroup of newborn survived in affected group, due to the smaller sample size after stratification.

**Table 3:** Stratification results of difference-in-difference analyses (DID) for indicators of economic status between affected households with newborn survived or not and comparison households (Mean, US $).

	Affected group with newborn survived (N = 102)			Comparison group (N = 346)					DID estimator 1 c	Affected group with newborn not survived (n = 81)			Comparison group (N = 346)			DID estimator 2 c
	Baseline	Follow-up	Δaffected^a^ (*p*-value)	Baseline		Follow-up		Δcomparison^b^ (*p*-value)	Δaffected-Δcomparison (*p*-value)	Baseline	Follow-up	Δaffected^a^ (*p*-value)	Baseline	Follow-up	Δcomparison^b^ (*p*-value)	Δaffected- Δcomparison (*p*-value)
Annual household income	2936	2758	−178 (0.435)^c^	3723		4538		815 (<0.001)	−993 (0.001)	2769	2869	100 (0.570)	3723	4538	815 (<0.001)	−715 (0.020)
per capita	682	750	68 (0.181)	838		1044		206 (<0.001)	−138 (0.027)	626	776	150 (0.610)	838	1044	206 (<0.001)	−56 (0.405)
Annual household expenditure	3013	2197	−816 (<0.001)	3211		3285		74 (0.429)	−890 (<0.001)	2628	2356	−272 (0.068)	3211	3285	74 (0.429)	−346 (0.095)
per capita	695	604	−91 (0.079)	738		776		38 (0.047)	−129 (0.020)	596	619	23 (0.610)	738	776	38 (0.047)	−15 (0.733)
Cumulated debts	2504	3899	1395 (0.013)	1956		2375		419 (0.002)	976 (0.088)	2315	3590	1275 (0.016)	1956	2375	419 (0.002)	856 (0.113)
Household wealth indices																
Index score of family property d	2.71	2.72	0.01 (0.836)	2.91	2.98		0.07 (0.382)		−0.06 (0.278)	2.65	2.63	−0.02 (0.658)	2.91	2.98	0.07 (0.382)	−0.09 (0.120)
Index score of live stocks e	2.60	1.96	−0.64 (0.135)	3.28	2.82		−0.46 (0.138)		−0.18 (0.782)	2.41	1.74	−0.67 (0.039)	3.28	2.82	−0.46 (0.138)	−0.21 (0.762)
Index score of basic living ability f	5.59	5.55	−0.04 (0.716)	5.77	5.82		0.05 (0.069)		−0.09 (0.331)	5.60	5.54	−0.06 (0.487)	5.77	5.82	0.05 (0.069)	−0.11 (0.015)

Affected group: households with a maternal death; Comparison group: matched households without maternal death.

a Δ_affected_ refers to the mean value of affected group in follow-up minus that at baseline.

b Δ_comparison_ refers to the mean value of comparison group in follow-up minus that at baseline.

c Numbers in parentheses are *P*-value.

d 1 score for each item with answer “have”, 0 score for each item with answer “don't have”. All 5 items were summed up. The items included (1) TV, (2) Cable TV, (3) Telephone, (4) Motorcycle, electrobicycle, or elctrotricycle, (5) Truck, tricar, or tractor.

e All 3 items were summed up. The items included (1) How many pigs are there in your family? (2) How many sheep is there in your family? (3) How many other live stocks are there in your family, such as cattle, horse, donkey, mule, etc?

f 1 score for each item with answer “yes”, 0 score for each item with answer “no”. All 6 items were summed up. The items included (1) Can your family have enough food to be not hungry? (2) Can your family eat meat, egg, or fish at least once every week? (3) Can your family buy enough clothes to keep warm in winter? (4) Can your family buy a piece of new clothes? (5) Can your family members go to the hospital when having indisposition? (6) Can your family buy some daily necessaries (e.g. Toilet paper, shampoo, soap, washing powder, etc)?

### Categories of annual income and expenditure

Table 4 showed the DID estimator of each category of annual income and expenditure at individual level. Total annual income included salary, family production, and income from transfer and estate. Although the mean differences between two groups were not statistically significant, income from salary, family production, and from transfer and estate had a relative decrease in the affected group compared with that in the comparison group during the following year after baseline survey (from US$ –13 to US$ –55).

**Table 4:** Categories of annual income and expenditure at individual level (Mean, US $).

	Affected group			Comparison group			DID estimator c
	Baseline	Follow-up	Δ_affected_ a (*p*-value)	Baseline	Follow-up	Δ_comparison_ b (*p*-value)	Δ_affected_-Δ_comparison_ (*p*-value)
Income							
Salary	308	388	80 (0.003)^c^	363	498	135 (<0.001)	−55 (0.124)
Family production	274	315	41 (0.164)	407	483	76 (0.001)	−35 (0.356)
Income from transfer and estate	76	58	−18 (0.154)	68	62	−6 (0.224)	−13 (0.451)
Expenditure							
Expenditure of production	103	95	−9 (0.165)	132	154	22 (0.167)	−31 (0.022)
Living expenses	500	480	−20 (0.364)	554	569	15 (0.790)	−35 (0.284)
Food	265	254	−10 (0.277)	288	310	22 (0.067)	−32 (0.037)
Clothes and commodity	34	22	−12 (0.032)	41	45	4 (0.127)	−16 (0.003)
Residence	34	35	2 (0.635)	43	41	−2 (0.696)	4 (0.617)
Transportation and phone	27	23	−4 (0.098)	37	40	2 (0.147)	−7 (0.022)
Health care	77	62	−15 (0.501)	71	66	−5 (0.673)	−10 (0.681)
Cigarette or alcohol	28	34	6 (0.001)	41	40	0 (0.913)	6 (0.014)
Other	104	100	−4 (0.263)	96	82	−13 (0.187)	10 (0.568)
Expenditure for transfer and estate or tax	48	38	−10 (0.140)	52	53	1 (0.586)	−12 (0.180)

Affected group: households with a maternal death; Comparison group: matched households without maternal death; DID: Difference-in-difference analyses.

a Δ_affected_ refers to the mean value of affected group in follow-up minus that at baseline.

b Δ_comparison_ refers to the mean value of comparison group in follow-up minus that at baseline.

c Numbers in parentheses are *P*-value.

Total annual expenditure included expenditure of production, living expense, and expenditure for transfer and estate or tax. Among the categories of living expenses, the mean expenditures on food, clothes and commodity, transportation and phone decreased significantly in affected households compared with that in the comparisons (*p = *0.037, 0.003, and 0.022, respectively). However, the mean expenditure on cigarette or alcohol in affected households significantly increased compared with that in the comparisons (*p = *0.014). During the interview, many male family members in affected households, especially the husbands, said that they tried to relieve their grief and loneliness by frequent drinking or smoking.

We also calculated proportions of household income and expenditure for each category. Results demonstrated lower proportion of clothes and commodity consumption but higher proportion of cigarette or alcohol consumption in affected households with statistical significance (see Table S1).

## Discussion

The baseline results of our study showed the immediate direct costs of maternal deaths were 37.0% of the household's annual income [6], which was approximately 4 times as high as the threshold for being considered catastrophic for the rural Chinese households [18], [19]. Based on the prospective cohort of the follow-up survey, the present study identified a strong and significant sustained impact of maternal death on the households' economy. In the year before maternal death or childbirth, the average annual household income and expenditure was US$2862 and US$2843 in affected group, and US$3723 and US$3211 in comparison group, while in the following year after maternal death or childbirth, the average annual household income and expenditure was US$2807 and US$2268 in affected groups, and US$4538 and US$3285 in comparison group, respectively.

The study used both subjective and objective indicators to comprehensively assess impact of maternal death on household economy. According to the results of subjective measure, sixty-three percent of households experiencing maternal death reported “household economy became worse” during the following year after the event. After adjusting for the impact of whether the newborn survived, maternal and household demographic characteristics, the risk of maternal death for self-reported “household economy became worse” increased from 4.07 (95% CI: 2.78–5.95) to 6.04 (95% CI: 3.61–10.08). The results of this subjective measure indicated an independent adverse effect of maternal death and further negative impact on the household economy if the newborn survived. As deduced by Basu [20], household experiencing a maternal death had lost its “home economist” (the women), and man was unaccustomed to managing the household budget and affairs.

The self-reported “household economy became worse” was verified by quantitative data with lower annual income and expenditure, higher debts, and lower wealth index, compared with the households having childbirth without maternal death. The results showed an increase in the annual income in affected group (Δaffected = US$104, *p = *0.004) but a reduction of annual expenditure (Δaffected = US$ –42, *p = *0.242) at individual level, which indicates efforts made by the affected households to cope with the economic burden of maternal death. However, these efforts were not sufficient to compensate for the expenditures associated with caring for the survived newborn. Borrowing money from relatives or friends was the major source of money to close the gap. Consequently, paying back the money would be a burden for them in a long run. Storeng et al. reported that debt and depletion of assets contributed to a cycle of debts with further selling assets and further loans to finance the obvious debts and to replenish lost tools, animals or stock for income-generating activities [8]. We performed stratified analyses and identified the impact of maternal death on economic indicators was not different among different socioeconomic groups, indicating the independent adverse impact of maternal death on household economy.

Unlike the newborn joining the family as a new member for a normal childbirth, a maternal death can result in loss of 1 or 2 members of a household because of high mortality of newborn. Results from stratification analysis of whether the newborn survived demonstrated multiple impact of the maternal death. Survived newborn in affect households may have dual impact on household income and expenditure. Household members had to stop part of income-generated work to take care of the baby. In addition, they reduced part daily consumption, except cigarette or alcohol, for raising the survived newborn by much more expensive complementary feeding instead of breast feeding.

### Comparison with other studies

Until now, there is no quantitative study on the sustained economic impact of maternal mortality at household level in the published or available gray literature that we have been able to retrieve. However, the economic impact of emergency obstetric care (near-miss events) has been explored in a few studies in recent years [7]–[9]. A study conducted in Mali [7] revealed adverse outcome of near-miss events, including loss of income, reduced food consumption and productivity, lack of food and in debt. A study conducted in Burkina Faso [8] reported that more frequent sale of assets, borrowing and slower repayment of debt in the year after near-miss. Both of these studies used small sub-sample analyses and showed the economic impact of near-miss on households, indirectly suggesting the potential impact of maternal death. Our quantitative study on maternal death directly showed its impact on households, including loss of income, reduced food consumption, and accumulated debts.

Most studies related to other illness have not examined economic impact of illness with prospective study design, except one study conducted in South African by Bachmann et al. This cohort study reported economic impact of HIV/AIDS was 13% reduction of annual household income in South Africa [21]. Results from our study showed that annual household income in the affected household reduced by 32.0% during the year after maternal death, and annual household expenditure in the affected group declined by 24.9%, which was similar to a reduction of 26% in the study by Bachmann et al [21]. In addition, we found the significantly reduced expenses of food, clothes and commodity, transportation and telecommunications at the individual level during the year after maternal death. These changes in the pattern of household expenditures were also observed by Over and Dayton, with households that had suffered an adult death exhibiting lower non-food expenditure per capita than non-affected households [22]. Except those reduced expenses, higher consumptions on cigarette or alcohol found by this study may further have adverse impact on the family members' health.

### Strength and limitation of the study

To our knowledge, the present study is the first prospective cohort study on the sustained economic impact of maternal death at household level. Although previous studies revealed financial hardship caused by emergency obstetric care, which is a useful complement to investigation of maternal death, this study showed the sustained economic impact of maternal death on household income and expenditure. It provided more direct and reliable evidences that maternal death would make adverse impact on household economy. Sustained worsening economy can impoverish households or push them further into poverty. It is urgent to increase funds for improving maternal health and for off-setting out of pocket expenditures for life-saving medical emergencies.

The sample size of our study (183 affected households and 346 comparison households) was relatively large compared to other, related studies. We enrolled not only the fatalities in hospitals but also those died at home, and the participants were selected from three provinces to represent lower, medium, and higher level of maternal mortality in rural China. In addition, the prospective study design with comparison households adds strength to the results.

In this study, both income and expenditure data was collected directly in each household. However, there is a debate on reliability of self-reported income and expenditure data [17], [23], [24]. Income reflects consumption opportunities; informants usually have better idea of average earnings of each family member, but can potentially conceal the facts for various reasons. Expenditure is an alternative poverty measure, but few informants record daily expenditure in detail and may omit a part. We used both of these measures in the study. Comparing annual income at individual level of comparison households in our study with the indicator of rural net income per capita in China published by National Bureau Statistics [14], [15], we found that data from comparison households in baseline study was not significantly different from the published data in 2009 (US$ 838 VS. 793, *p = *0.210), and data from comparison households in follow-up study was also not significantly different from the published data in 2011 (US$ 1044 VS. US$ 1073, *p = *0.620). These results increased the confidence in the validity of our findings.

The limitation of this study is that we didn't investigate the mechanism of how maternal death affected household economy. Further studies should focus on the pathway from maternal death to the worsening household economy, providing more implications and recommendations for social assistance interventions.

### Implications for policies

The study has several implications for health and social policies. First, catastrophic costs related to maternal death and its adverse lasting impact on household economy justify efforts to improve coverage of preventive measures on maternal mortality. Although the international community has a strong commitment to improve maternal-newborn ill-health (MNIH) in developing countries, direct funding for reproductive health has not increased in recent years, and in many countries it has declined relative to demand for services. Increase of funding on maternal mortality prevention could contribute to poverty reduction [5].

Second, financial supports to cope with high direct costs of maternal death is particularly important, such as expanding the coverage of financing systems, and increasing the reimbursement to protect households experienced maternal death with catastrophic direct costs. One point to be discussed is whether to develop financing mechanisms which eliminate all medical expenses and cover the majority of women treated with emergency obstetric care (EMOC), or to protect and subsidy the minority of households that have suffered from catastrophic costs caused by maternal death. Until now, maternity insurance existed in several provinces of China, providing one-off subsidy for funeral expenses and pension to the family experienced maternal death by using surplus fund. This subsidy is a feasible measure to reduce the economic burden of maternal death for each household. Insurance benefit packages are capped at a maximum amount, which is insufficient for end-of-life medical emergency expenses – for maternal health as well as other conditions. Although extraordinary measures are put into place by local hospitals and government to compensate the families, these measures are ad hoc and, as we saw in this study, insufficient.

Third, the results showed a reduction of both income and expenditure of family production, even lower food consumption per capita in affected households. It may have an adverse impact on physical health status of family members. Ainsworth and Gertler indicated that children who lost their mothers were much more likely to be stunted and maternal death increases the probability of both child death and being malnourished than children with their parents alive [25], [26]. We suggested giving credit to the affected households in the short term to increase labor and land productivity, and consequently avert potential household food insecurity and health problems.

### Conclusion

Compared with childbirth, maternal death had adverse impact on household economy, including decreased income and expenditure, increased debts, changed expenditure pattern, and higher risk of self-reported “household economy became worse”. The present study is the first prospective cohort study providing more direct and reliable evidences for adverse economic impact of maternal death at household level. Sustained worsening economy can impoverish households or push them further into poverty. Increase of funding on maternal mortality prevention could contribute to poverty reduction.

## Supporting Information

Table S1 **Proportions of household income and expenditure for each category.**
(DOC)
