# Supplementary material for: Economic Impact of Maternal Death on Households in Rural China: A Prospective Cohort Study
Source: PLoS One. 2013 Oct 24;8(10):e76624. doi: 10.1371/journal.pone.0076624 (PMC3811988; doi:10.1371/journal.pone.0076624)
Supplement: Table S1 — Proportions of household income and expenditure for each category. (DOC) [file pone.0076624.s001.doc]

Table S1 Proportions of household income and expenditure for each category

|  | Affected group | | |  | Comparison group | | |  | DID estimator ^c^ |
| --- | --- | --- | --- | --- | --- | --- | --- | --- | --- |
|  | Baseline | Follow-up | Δ_affected_^a^ |  | Baseline | Follow-up | Δ_comparison_^b^ |  | Δ_affected_-Δ_comparison_ |
| Income |  |  |  |  |  |  |  |  |  |
| Salary | 0.40 | 0.43 | 0.03  (0.225) |  | 0.44 | 0.49 | 0.05  (<0.001) |  | -0.02  (0.250) |
| Family production | 0.49 | 0.47 | -0.02  (0.353) |  | 0.48 | 0.45 | -0.03  (0.007) |  | 0.01  (0.448) |
| Income from transfer and estate | 0.11 | 0.10 | -0.01  (0.547) |  | 0.08 | 0.06 | -0.02  (0.007) |  | 0.01  (0.419) |
| Expenditure |  |  |  |  |  |  |  |  |  |
| Expenditure of production | 0.17 | 0.16 | -0.01  (0.408) |  | 0.15 | 0.17 | 0.02  (0.057) |  | -0.03  (0.098) |
| Living expenses | 0.77 | 0.79 | 0.02  (0.159) |  | 0.78 | 0.77 | -0.01  (0.103) |  | 0.03  (0.030) |
| Food | 0.44 | 0.44 | 0.00  (0.953) |  | 0.43 | 0.44 | 0.01  (0.360) |  | -0.01  (0.614) |
| Clothes and commodity | 0.05 | 0.04 | -0.01  (0.027) |  | 0.05 | 0.06 | 0.01  (0.462) |  | -0.02  (0.020) |
| Residence | 0.06 | 0.07 | 0.01  (0.483) |  | 0.06 | 0.06 | 0.00  (0.334) |  | 0.01  (0.238) |
| Transportation and phone | 0.04 | 0.04 | 0.00  (0.347) |  | 0.05 | 0.05 | 0.00  (0.157) |  | 0.00  (0.799) |
| Health care | 0.08 | 0.08 | 0.00  (0.812) |  | 0.08 | 0.07 | -0.01  (0.381) |  | 0.01  (0.482) |
| Cigarette or alcohol | 0.05 | 0.06 | 0.01  (0.012) |  | 0.06 | 0.05 | -0.01  (0.141) |  | 0.02  (0.005) |
| Other | 0.05 | 0.06 | 0.01  (0.396) |  | 0.05 | 0.04 | -0.01  (0.185) |  | 0.02  (0.956) |
| Expenditure for transfer and estate or tax | 0.06 | 0.06 | 0.00  (0.788) |  | 0.06 | 0.06 | 0.00  (0.927) |  | 0.00  (0.785) |

Affected group: households with a maternal death; Comparison group: matched households without maternal death; DID: Difference-in-difference analyses

^a^Δ_affected_ refers to the mean value of affected group in follow-up minus that at baseline.

^b^Δ_comparison_ refers to the mean value of comparison group in follow-up minus that at baseline.

^c^ Numbers in parentheses are *P*-value.
